# Efficacy of Group Music Therapy Based on Emotion-Regulation Skills on Male Inpatients With Alcohol Dependence: A Randomized, Controlled Pilot Trial

**DOI:** 10.3389/fpsyg.2021.697617

**Published:** 2021-10-28

**Authors:** Yi Huang, Xu Chen

**Affiliations:** ^1^School of Humanities and Social Sciences, Binzhou Medical University, Yantai, China; ^2^Department of Substance Abuse, Shandong Mental Health Center Affiliated to Shandong University, Jinan, China; ^3^Department of Substance Abuse, Shandong Mental Health Center Affiliated to Jining Medical University, Jinan, China

**Keywords:** alcohol dependence, intensive group psychotherapy, music therapy, emotion regulation, inpatient treatment, feasibility

## Abstract

**Objective:** This study aimed to determine the benefits and feasibility of using group music therapy based on emotion-regulation skills to treat male inpatients with alcohol dependence (AD).

**Methods:** We recruited male inpatients with alcohol dependence and randomly assigned those eligible for enrollment to either the study group or the control group. The study group received group music therapy along with treatment-as-usual (TAU), while the control group received only treatment-as-usual. Primary outcomes, including anxiety levels, sleep quality, and alcohol craving, were assessed at baseline and after 2 weeks of treatment. Secondary outcomes included feasibility measures such as dropout rates. We evaluated the acceptability of group music therapy based on semi-structured interviews and feedback from patients and therapists.

**Results:** The average attendance rate of the study group patients who underwent group music therapy was 70.77%, and the drop-out rate was 7.69%. Based on intention-to-treat analysis, we found no differences in baseline assessments (*p* > 0.05). Assessment after 2 weeks of treatment showed that study group patients were less anxious, slept better, and had reduced alcohol cravings than control group patients. However, these differences were not statistically significant. Participants reported that group music therapy made them feel more relaxed and improved their mood.

**Conclusion:** Group music therapy based on emotion-regulation skills is feasible with potential for efficacy and can be used to treat men with alcohol dependence in a closed inpatient environment. Further long-term research is required to gain a better understanding of the efficacy of using group music therapy to treat alcohol dependence.

## Introduction

Alcohol is a major risk factor associated with injuries and poor health (Astrup and Estruch, 2019). Alcohol-related disorders have a severe impact on public health in countries across the world, resulting in 3 million deaths each year globally and contributing to disability and poor health in millions of people. Based on 12-month prevalence estimates, substance use disorders, including alcohol use disorder, have been reported as the third most common mental disorders observed in China (Huang et al., 2019). In fact, alcohol use disorder is the most prevalent mental disorder observed in patients from Shandong Province in China (Chen, 2019). Compared to other substances, alcohol consumption is prevalent among the Chinese population, especially because it can be obtained easily. Apart from its serious consequences for individual health and safety, alcohol use disorder can pose a severe social and economic burden on patients and their families.

Current treatments for alcohol dependence (AD), a subtype of alcohol use disorder, include drug therapy, physical therapy, and psychotherapy. Given the complexity of AD, its associated comorbidities and substantial recurrence, the recommended psychotherapeutic treatments are comprehensive, multi-dimensional psychological intervention models, including motivational enhancement therapy, cognitive behavioral therapy, and 12-step facilitation therapy (Yi et al., 2019). However, these are primarily conversation-based therapies that focus on cognition and behavior.

Regardless of the treatment received, AD patients show high relapse rates, and even with evidence-based treatment, nearly 67% of AD patients drink again after 1 year of treatment (Coriale et al., 2018). However, increased cravings, a risk factor for redrinking (Pombo et al., 2016), have been associated with impaired emotional regulation, and they usually manifest as increased negative emotions and decreased positive emotions (Khosravani et al., 2017; Jansen et al., 2019). In this regard, researchers believe that relapse rates among AD patients can be reduced by improving their emotional state (Zhu et al., 2019), or by ensuring their continued participation in treatment programs (Megranahan and Lynskey, 2018).

Alcohol dependence patients typically experience anxiety, irritability, insomnia, and other conditions related to their emotional state (Chakravorty et al., 2016). Anxiety and insomnia often interact with drinking behavior (Chaudhary et al., 2020), and studies have shown that the thalamus has an important role in this interaction (Zhornitsky et al., 2018; Liu et al., 2019). At the same time, the thalamus plays a role in the impact of music on humans (Koelsch, 2014; Linnemann et al., 2015): listening to music activates the interconnected networks of the subcortical and cortical brain structures, including the hypothalamus (Menon and Levitin, 2005). Therefore, music therapy can be used to improve mental health and well-being by alleviating negative emotions (Aalbers et al., 2017; Feng et al., 2018; Ghetti et al., 2020).

At present, the number of randomized controlled studies on the use of music therapy in AD patients is very limited, and most of the relevant studies have included patients with multiple types of substance use disorders simultaneously (Huang and Chen, 2021). Among them, in terms of time-frequency, most of them used a weekly setting of 45 min each session. In terms of the choice of music therapy method, it was mainly based on cognition or behavior, such as composing lyrics by discussing a given topic thus enhancing motivation for treatment (Silverman, 2019); temporarily alleviating withdrawal and craving by discussing lyrics in songs (Silverman, 2016); and enhancing effects of systemic desensitization by listening to New Age music (Stamou et al., 2017). Psychological interventions in AD that directly intervene in emotions and feelings have been neglected.

However, in clinical practice, it is more important to prioritize the emotional health of patients during rehabilitation than to focus on other efficacy measures (Witkiewitz et al., 2019). Therefore, it is critical to explore psychotherapeutic methods that use a direct intervention approach to improve emotions in patients with AD. A combination of music therapy and emotion-regulation skills may be effective at treating AD patients because it takes emotional feelings as the starting point to influence drinking behavior and adverse coping patterns.

In addition, the proportion of men among AD patients in China is 16–17 times higher than that of women (Huang et al., 2019). AD is essentially a male phenomenon in China (Cheng et al., 2015; Phillips et al., 2017), and nearly all of those requiring hospitalization for AD are male. So this study chose to focus only on male patients.

The purpose of the present study was to develop a psychotherapy program that uses emotional perception as an entry point for the treatment of patients suffering from AD. Here we evaluate the feasibility and efficacy of using intensive group music therapy based on emotion-regulation skills to treat Chinese male inpatients with AD.

## Methods

### Study Design

This prospective randomized controlled trial was performed based on the guidelines recommended in the Consolidated Standards of Reporting Trials (CONSORT) Statement. We used Zelen’s design (Zelen, 1979) to compare the efficacy of treating AD patients with treatment-as-usual (TAU) alone or combined with group music therapy. Zelen’s design, also known as a post-randomized consent design, is a trial method of randomizing participants before acquiring consent in order to enhance recruitment to clinical trials (Adamson et al., 2006). Based on the results of previous studies and pretests, the calculated sample size was set at 12 per group to detect the clinical effect of the two groups in terms of anxiety levels, sleep quality, and alcohol craving (two-tailed test with α = 0.05, power 80% and anticipating drop-out of 20%).

### Patients

We recruited a convenience sample of male patients with AD who were hospitalized in the Substance Abuse ward of the Shandong Mental Health Center between August and December 2020. All patients were diagnosed and screened by three experienced psychiatrists.

Patients who met the eligibility criteria were numbered according to order of admission and randomly assigned to the study group or the control group using Microsoft Office Excel 2007 (Microsoft, Redmond, WA, United States). Informed consent was obtained individually for each group: patients in the control group were asked to provide consent on their willingness to participate in the study, and patients in the study group were asked to provide consent on their willingness to accept group music therapy along with TAU.

### Eligibility Criteria

We included all patients who fulfilled the diagnostic criteria for AD outlined in the International Classification of Diseases (ICD)-10, who had a Michigan Alcoholism Screening Test (MAST; Wang et al., 1999) score ≥6, and who had mild physical withdrawal symptoms or no such symptoms [score ≤9 on the Clinical Institute Withdrawal Assessment for Alcohol-Revised (CIWA-Ar; Zhuo et al., 2010)], such that they could participate fully in the study. Furthermore, we included only patients who had more than 6 years of education, those who were able to understand and cooperate with relevant treatment and evaluation procedures, and those who provided written informed consent to participate in the study.

**FIGURE 1:**
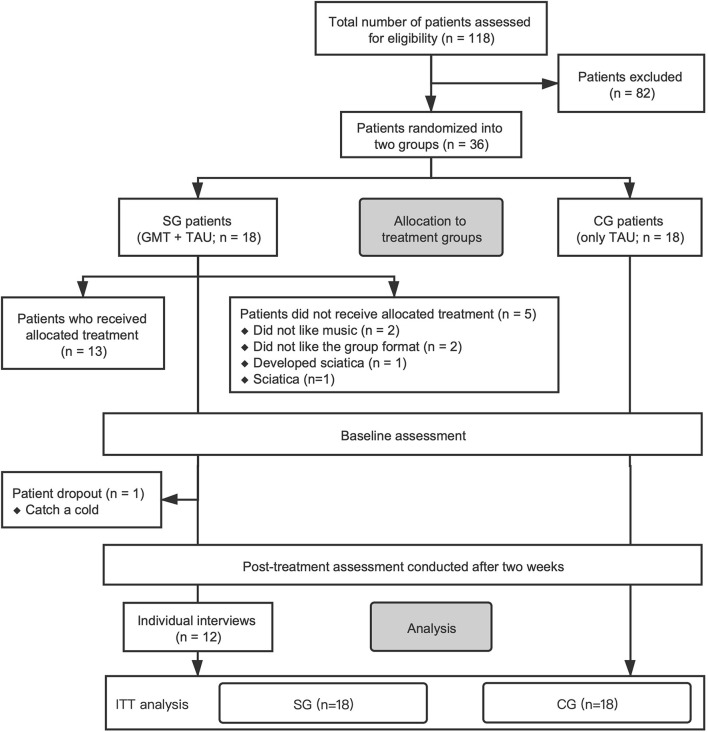
Consolidated Standards of Reporting Trials (CONSORT) participant flowchart depicting the patient selection process. SG, study group; CG, control group; GMT, group music therapy; TAU, treatment-as-usual; ITT, intention-to-treat analysis.

We excluded AD patients who suffered from severe physical diseases and psychotic symptoms, those who reported having serious suicidal, self-injury, or injury tendencies, as well as those who reported a dependence on any substances other than alcohol. Details on the patient selection process are depicted in Figure 1.

Drop-out Criteria includes: (1) patient-initiated request to dropout this study; (2) missing interviews; (3) the physician in charge and/or music therapist determines that it is inappropriate to continue (if there are serious complications or comorbidities; Severe adverse events).

### Therapeutic Intervention

#### Treatment-as-Usual

Patients in both study group and control group received routine TAU involving drug therapy, psychological, and health education treatment. All TAU treatments were administered to patients by the clinical team in the addiction ward of the Shandong Mental Health Center.

#### Group Music Therapy

The group music therapy protocol used in this study was designed by a licensed psychotherapist trained in music therapy and dialectical behavior therapy (DBT), after considering the status of the AD patients enrolled. This protocol consisted mainly of receptive music therapy, along with an emotion-regulation module (Linehan, 2015a,b) derived from DBT. Specifically, it includes receptive music therapy approaches such as musical relaxation, guided or unguided musical imagery, and song discussion (Gardstrom et al., 2015), as well as emotion regulation skills such as breathing relaxation, mindfulness practice, observing emotions and opposite action (Linehan, 2015a).

The process of expressing and experiencing emotions is a two-way street (Shapiro, 2019). Expressions, postures or movements not only help us to express and understand our own emotional feelings and those of others, but different expressions, postures or movements also affect our own current feelings in turn. Therefore, this intervention protocol integrates music listening, focusing on feelings and emotion regulation techniques.

On the choice of music content, emotion as the core, a variety of different rhythms, speed, melodies, harmonies, arrangements, and styles of music can be chosen. According to the theme of each session (such as fear, anger, sadness, etc.) in a relatively safe range, the various emotions of patients can be fully aroused, and to do further work, such as describing one’s feelings at the moment with weather or colors, or experience their own emotional feelings and changes under different expressions and postures.

Each session of the group music therapy followed the same basic structure and treatment strategy: warm-up, review of previous session and homework, music listening, focusing on feelings (Gendlin, 1996), emotion-regulation skills, and homework assignments for the next session.

Of the 18 randomly assigned to study group, 5 did not receive the new protocol of group music therapy in combination with TAU; they actually received TAU only, but the primary outcome indicator for these 5 was counted as part of the study group, according to intention-to-treat analysis. Thus, these 5 patients and all patients randomly assigned to control group (*n* = 18) only received routine treatment. The remaining participants who actually experienced group music therapy (*n* = 13) received TAU along with 60 min of group music therapy each day over a period of two consecutive weeks (weekends were excluded; total 10 sessions). group music therapy was led by the same therapist in closed groups of 6–8 patients in order to avoid confounding due to different therapists or treatment content.

### Data Collection and Outcomes

We collected baseline data on age, ethnicity, years of education, marital status, employment status, economic status; AD diagnosis, previous medical history, family history of alcohol-related disorders, number of previous hospitalizations, use of other substances; as well as experience with music (casual listening, playing an instrument, playing in a band) and preferred genre of music.

The primary outcomes were changes in anxiety levels, sleep quality, and alcohol craving level between baseline and after 2 weeks of treatment. The secondary outcome was the feasibility of using intensive group music therapy based on emotion-regulation skills to treat male AD patients in a closed inpatient setting (see section “Feasibility”). These results were obtained based on clinical observations and semi-structured interviews. Each patient was interviewed individually in a relatively private area, and interviews lasted up to an average of 45 min.

### Assessments

Baseline assessment was performed immediately after enrollment. The post-treatment assessment was performed within 24 h after the end of the 10-session group music therapy intervention. Experienced psychiatrists who were blinded to the purpose and design of the study collected all demographic data and performed examinations. Additionally, during the initial stages of the group music therapy intervention process, study group patients underwent a music evaluation, which was performed by the therapist who led their music therapy group.

#### Clinical Outcomes

##### Michigan alcoholism screening test

The Chinese version of MAST is a 24-item scale, which is used to assess the severity of AD (Wang et al., 1999). It has good test-retest reliability (*r* = 0.95), and its Cronbach’s alpha values range from 0.83 to 0.90 (Hsueh et al., 2014). These 24 items include interpersonal relationships, work performance, health status, legal issues, and family problems. For example, “Are you always able to stop drinking when you want to?” and “Have you ever lost a job because of drinking?” (Selzer, 1971; Wang et al., 1999). Scoring was performed using the simple summation method (Skinner, 1982), which means each positive response was scored as 1, otherwise scored as 0. So the total score ranged from 0 to 24, with higher scores indicating more severe AD. This scale was used in this study primarily to screen AD patients who met the inclusion criteria.

##### Clinical institute withdrawal assessment for alcohol-revised

The CIWA-Ar is a 10-item examiner-rating scale, which has good reliability and validity. Cronbach’s alpha value of this scale is 0.85, and the ICC value was 0.73. The ten items correspond to ten symptoms, including nausea, vomiting, anxiety, disturbances of orientation, and so on. For example, “Do you feel sick to your stomach and have you vomited?” and “Do you feel nervous?” (Sullivan et al., 1989). Each symptom is scored on a scale of 0 to 7. The total score is the sum of the scores of ten items, with the higher score meaning the more severe withdrawal syndrome (Zhuo et al., 2010). In this study, participants were independently screened by three trained psychiatrists in order to exclude patients who were still in the acute withdrawal period.

##### Self-rating anxiety scale

The self-rating Anxiety Scale (SAS) is a 20-item scale, which covers a variety of anxiety symptoms. For example, “I feel afraid for no reason at all” and “My arms and legs shake and tremble” (Zung, 1971). Responses are given on a 4-point scale which ranges from 1 (none, or a little of the time) to 4 (most, or all the time). The sum of the scores of the 20 items is the raw score, ranging from 20 to 80. The index score is used as the final score, which is an integer equal to 1.25 times the raw score, and higher scores indicate more severe anxiety symptoms. The reliability and validity of this scale were good, where Cronbach’s alpha value is 0.82. The Pearson correlation coefficient was 0.365 and the Spearman correlation coefficient was 0.341 using Hamilton Anxiety Scale as a reference (Wang et al., 1999).

##### Pittsburgh sleep quality index

Sleep quality was assessed using the Pittsburgh Sleep Quality Index (PSQI), a 24-item self-rating scale. These items measure sleep disorders in seven areas, including subjective sleep quality, sleep duration, use of sleep medication, and so on. For example, “Cannot get to sleep within 30 min” and “Wake up in the middle of the night or early morning.” Scoring on the items ranges from 0 (none) to 3 (three or more times). These subscales are summed to produce a global PSQI score, which ranges from 0 to 21. Higher global scores on the PSQI indicate worse sleep quality. The overall reliability coefficient of the PSQI was 0.82–0.83. The test-retest reliability was good (Buysse et al., 1989; Tsai et al., 2005).

##### Craving-visual analog scale

The levels of alcohol craving were assessed using the Craving-Visual Analogue Scale (CVAS), which is a self-rating scale. The amount of alcohol craving that a participant felt ranged across a continuum from 0 (no craving) to 10 (strong craving). The CVAS score was determined by measuring from the left-hand end of the line to the point that the participant marked (Hu and Chen, 2020). Higher scores indicate a more severe craving for alcohol.

These self-rating scales are reported to have high confidence validity and open accessibility (Zhang and He, 2015). Test-retest reliability was verified by randomly selecting nine patients from each group and re-measuring their clinical indicators 4 h after each evaluation: different subsets of nine patients were re-evaluated after the baseline and after 2 weeks of treatment.

#### Feasibility

The feasibility of using group music therapy based on emotion-regulation skills to treat AD patients was assessed based on consent rate, session attendance, rate of compliance with and acceptance of treatment, completion of the assessment at 2 weeks, adverse events, feedback from patients on the study and treatment protocols, and informal feedback from health care professionals.

Individual patient feedback was collected based on semi-structured interviews conducted after group music therapy intervention. Patients were invited to describe their experience and feelings, give specific feedback on effective and ineffective sessions, suggest potential improvements in the intervention protocol, as well as report their views on the acceptability, suitability, and efficacy of group music therapy.

### Data Analysis

#### Quantitative Data

For each of the outcome variables, we compared the average difference between the control group and study group conditions in terms of improvements in outcome measures between baseline and after 2 weeks of treatment. The comparison between the two groups was made on an intention-to-treat basis. For intra-group comparisons, a Wilcoxon signed-rank test was used. For comparison between groups, a Mann–Whitney *U* test was used.

#### Qualitative Data

The interview transcripts were imported into MAXQDA 2020 and coded and analyzed by one researcher to ensure consistency. Steps included reading the interview transcripts verbatim, initial coding, extraction of thematic frames, asking the interviewees for confirmation, and textual restructuring.

## Results

### Quantitative Data Analysis

#### Patient Characteristics

We recruited a total of 118 AD patients who were hospitalized in the addiction ward of the Shandong Mental Health Center between August and December 2020 (Figure 1). After a detailed assessment based on eligibility criteria, we excluded patients who, for example, had less than 6 years of education, suffered from severe physical illness, were accompanied by psychotic symptoms, were in acute withdrawal, or had a tendency to self-injure or injure others. Ultimately, 36 men were enrolled in the randomized controlled trial and allocated to two treatment groups: the study group (*n* = 18), which was to receive group music therapy and TAU; or the control group (*n* = 18), which received TAU alone. Only 13 study group patients ultimately consented to receive group music therapy intervention (Figure 1).

Baseline assessments of demographic and clinical characteristics indicate no significant differences between the two groups in age, years of education, duration of AD, number of previous hospitalizations caused by AD, or duration of current hospitalization (*p* > 0.05; Table 1). All study group and control group patients were right-handed and from the Han Chinese ethnic group.

**TABLE 1:** Baseline differences in demographic variables and clinical measures.

Characteristic	Control group (*n* = 18)	Study group (*n* = 18)	*p*
**Age** (years)	40.22 ± 6.11	41.39 ± 5.08	0.54
**Education** (years)	10.50 ± 2.79	10.17 ± 2.71	0.72
**Marital status**			1.00
Married	16 (88.89)	16 (88.89)	
Divorced	2 (11.11)	2 (11.11)	
**Employment status**			0.31
Employed	18 (100)	17 (94.44)	
Unemployed	0	1 (5.56)	
**Duration of AD** (years)	8.28 ± 6.12	9.28 ± 5.10	0.72
**Number of previous hospitalizations due to AD**	2.94 ± 5.00	2.50 ± 1.20	0.91
**Duration of current hospitalization** (days)	46.19 ± 20.60	44.31 ± 20.62	0.80
**Past history** (yes)			
Hypertension	7 (38.89)	7 (38.89)	1.00
Diabetes	0	1 (5.56)	0.31
Heart disease	0	2 (11.11)	0.15
Depressive disorder	1 (5.56)	2 (11.11)	0.55
Bipolar disorder	1 (5.56)	1 (5.56)	1.00
Other conditions	13 (72.22)	15 (83.33)	0.42
**Family history** (yes)			
Mental disorder	2 (11.11)	1 (5.56)	0.55
Alcoholism	6 (33.33)	12 (66.67)	0.09
**MAST scores**			0.54
6–13	10 (55.56)	8 (44.44)	
14–20	7 (38.89)	9 (50.00)	
21–24	1 (5.56)	1 (5.56)	

*AD, alcohol dependence; MAST, Michigan Alcoholism Screening Test.*

*All values are mean ± SD, or *n* (%), unless otherwise specified.*

#### Clinical Indicators

Baseline measurements of clinical indicators showed no significant differences between the two groups (*p* > 0.05). An assessment conducted after 2 weeks indicated that study group patients tended to show greater improvement in clinical indicators than control group patients (SAS-*D*-value: 9.00 > 6.50; PSQI-*D*-value: 3.50 > 1.50). However, this difference was not statistically significant (*p*^3^ > 0.05; *p*^4^ > 0.05).

**FIGURE 2:**
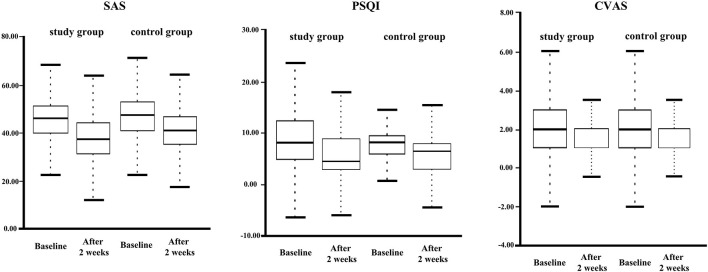
Box-polt of inter- and intra-group comparisons at baseline and after 2 weeks of treatment.

Intra-group comparisons showed that sleep quality and anxiety levels improved significantly in both groups (Figure 2 and Table 2). Although there was a reduction in the alcohol craving index in both groups, this was not significant. Our evaluation of SAS, PSQI, and CVAS showed an intra-group correlation coefficient >0.8 and good test-retest reliability.

**TABLE 2:** Inter- and intra-group comparisons of self-rating assessments of clinical indicators at baseline and after 2 weeks of treatment.

Scale	Study group (*n* = 18)			*p* ^1^	Control group (*n* = 18)			*p* ^2^	*p* ^3^	*p* ^4^
	M^1^ (P25, P75)	M^2^ (P25, P75)	D^1^		M^3^ (P25, P75)	M^4^ (P25, P75)	D^2^			
**SAS**	46.00(39.8,51.3)	37.00(31.0,44.3)	9.00	0.01**	47.50(40.8,53.0)	41.00(35.0,47.0)	6.50	0.04*	0.47	0.36
**PSQI**	8.00(4.8,12.3)	4.50(3.0,9.0)	3.50	0.00**	8.00(5.8,9.3)	6.50(3.0,8.0)	1.50	0.00**	0.81	0.79
**CVAS**	2.00(1.0,3.0)	2.00(1.0,2.0)	0.00	0.09	2.00(1.0,3.0)	2.00(1.0,2.0)	0.00	0.12	0.95	0.79

*p < 0.05, **p < 0.01.

*M^1^ and M^3^, Baseline; M^2^ and M^4^, After 2 weeks; *D*^1^ = M^1^−M^2^; *D*^2^ = M^3^−M^4^.*

*CVAS, Craving-Visual Analog Scale; PSQI, Pittsburgh Sleep Quality Indexes; SAS, Self-rating Anxiety Scale.*

**p*^1^, Comparison of study group values; *p*^2^, Comparison of control group values; *p*^3^, Comparison of inter-group baseline values; *p*^4^, Comparison of inter-group values measured after 2 weeks of treatment.*

### Qualitative Data Analysis

#### Assessment of Treatment Feasibility

Our findings indicate that it is feasible to treat male patients with AD using group music therapy in a closed inpatient setting. For the results of randomization, participants’ overall consent was 86.11% (control group = 100%, study group = 72.2%), which was significantly higher than other similar studies (56–77%; Bradt et al., 2016; Low et al., 2020). Of the 13 patients in the study group, 9 attended at least 7 of the 10 group music therapy sessions. Overall participation in the group music therapy sessions was high, with an average attendance rate of 70.77%, similar to previous studies (Muld et al., 2016). The main reasons for failure to attend group music therapy sessions were discharge from the hospital and scheduling conflicts with other treatments or examinations. The retention rate in the study group was extremely high (12, 92.3%), similar to one previous study (Trimmer et al., 2018) but much higher than several other studies (73–84%; Wood et al., 2018; Domínguez-Chávez et al., 2019): only 1 patient who was suffering from a cold dropped out due to physical discomfort. Overall treatment compliance was good. No adverse events occurred throughout the implementation of group music therapy.

#### Assessment of Treatment Acceptability

We received positive feedback on the benefits and acceptability of group music therapy from the study group patients and medical staff. Patients who received group music therapy indicated that all interventions were easy to understand and to participate in. Participants were “satisfied” or “very satisfied” with the content and setting of the group music therapy as well as with the group music therapy leader (Table 3).

**TABLE 3:** Qualitative assessment of group music therapy administered to 12 male inpatients with alcohol dependence (AD).

Categories	Theme	Subtheme	*n*	%
**Changes and benefits**	Motivation and willingness to accept treatment	Improved confidence and motivation levels in treatment and rehabilitation	12	100%
		Increased willingness to continue to participate in similar treatment activities, during hospitalization or after discharge	12	100%
		No resistance or conflict regarding further hospitalization and treatment	6	50%
	Emotional and mental status	More comfortable and improved mood	12	100%
		Increase in positive emotions experienced in daily life	7	58.33%
		Decrease in negative emotions experienced in daily life	4	33.33%
		No significant change in emotional experience	2	16.67%
		Improvement in frame of mind	5	41.67%
	Expression of emotions and interpersonal communication	Reduced irritability, emotions are expressed more casually, with less irritability and impulsiveness	2	16.67%
		Increased willingness to communicate; no longer shy	4	33.33%

**Content of group music therapy**	Beneficial exercises	Breathing relaxation and muscle relaxation	12	100%
		Guided musical imagery	12	100%
		Musical experience with different emotional themes	9	75%
		Focus on feelings and maintaining mindfulness	6	50%
	Skills taught - ease of use and willingness to use	Breathing relaxation	11	91.67%
		Progressive muscle relaxation	6	50%
		Mindfulness exercises	5	41.67%
		Adjustment of expression or posture	4	33.33%
		Emotional emergency box	3	25%
	Acquisition of new coping methods	Learning several coping strategies, other than drinking, in the face of emotional distress, stress, cravings, and impulses	10	83.33%

**Satisfaction**	Content and protocol of group music therapy	Very satisfied	9	75%
		Satisfied	4	33.33%
		Indifferent	0	–
		Dissatisfied	0	–
		Very dissatisfied	0	–
	Coordinator of group music therapy and implementation style/method	Very satisfied	12	100%
		Satisfied	1	8.33%
		Indifferent	0	–
		Dissatisfied	0	–
		Very dissatisfied	0	–

Among them, all of these participants indicated that group music therapy had improved their confidence and motivation levels in treatment and rehabilitation; they were very willing to continue to participate in similar treatment activities, during hospitalization or after discharge; their mood was more comfortable and had improved after participating in it. For the content setting of group music therapy, participants agreed that breathing relaxation, muscle relaxation, and guided musical imagery were the most helpful parts; for the coping skills and methods taught during group music therapy, breathing relaxation was considered the most useful and easy to use (11/12). In addition, the majority of participants (10/12) reported that group music therapy allowed them to discover increasingly more suitable options for dealing with emotional distress, stress, cravings, and impulses, such as listening to music in their established functional singing lists, relaxing, focusing on their feelings and maintaining mindfulness about them rather than drinking.

## Discussion

As a useful attempt to apply intensive group music therapy based on emotion-regulation skills to male AD inpatients, this study received very positive feedback from group music therapy participants. The acceptability and feasibility of this protocol are notable, and the possible reasons are as follows. In a closed hospital environment, relatively monotonous hospitalization makes AD patients prone to boredom and tediousness. Group music therapy not only takes participants’ attention away from negative feelings and enriches their hospitalization life, but it also provides them with a safe zone for emotional release, thus improving their overall emotions. The group music therapy in this study not only utilized the intensive group format with time and frequency settings, but its content also included emotion-regulation skills from DBT. This increases the practicality and operability of group music therapy, while also keeping the group interested, which makes the therapy more acceptable to them. Consequently, group music therapy should be widely promoted as an interesting, practical treatment for AD inpatients.

In terms of the primary outcome measures (anxiety level, sleep quality, craving level), the results of this study were similar to previous similar studies (Hohmann et al., 2017; Mathis and Han, 2017). However, the difference in efficacy between study group and control group was not statistically significant. The reasons may be as follows.

One reason may be the total time of group music therapy and the duration of effect maintenance. On the one hand, AD patients require an acute withdrawal period of approximately 2 weeks at the beginning of hospitalization. This allows them to receive group psychotherapy, such as group music therapy done for a shorter period during their hospitalization compared with other patients (Wang et al., 2018). Compared to the common weekly group therapy frequency, intensive group therapy five times a week minimizes the discontinuity caused when participants drop out (Yalom, 2015). On the other hand, considering that the minimum length of stay for AD patients in our hospital is usually around 25 days, we set the treatment time with group music therapy to 2 weeks to ensure that they can participate. However, this may lead to weak consolidation efficacy, as the efficacy of group music therapy usually increases with the number of sessions (Megranahan and Lynskey, 2018). Additionally, there may be some timeliness in the efficacy of group music therapy. Although the immediate evaluation after each treatment will indicate a more significant effect than later assessments (Cevasco et al., 2005; Volpe et al., 2018), the primary outcome measures were evaluated only at baseline and after 2 weeks of treatment in the present study in order to avoid test fatigue. Consequently, the possibility of efficacy subsidence cannot be ruled out (Taets et al., 2019).

The second reason may be the difficulty of efficacy evaluation based on the subjective feelings of patients. Due to the use of a self-rating scale for primary outcome indicators in the present study, numerous participants (especially the control group) may deliberately hide their real situation so as to prove to their doctors that they have recovered and can be discharged as soon as possible. Furthermore, for the subjective feelings of sleep quality and anxiety level, some participants may have inaccurate self-perception. For example, although they actually sleep at night, they may report that they are not sleeping well, or vice versa. This may lead to no significant observed difference between the two groups. Nevertheless, given the limited choice of assessment tools, such as the lack of clinical assessment tools for cravings, the CVAS was applied because it is currently widely used and recognized as an effective and reliable assessment tool (Hu and Chen, 2020). We chose the PSQI because it is also a widely used and authoritative tool for the evaluation of sleep quality. However, the PSQI is not sensitive to changes in sleep in the short term (less than 1 month), which may also be a reason for the insignificant difference between the two groups (Buysse et al., 1989). Subsequent studies may wish to explore more objective evaluation methods of biological indicators. Although there was no significant difference between the two groups in terms of sleep quality or anxiety, both groups demonstrated significant improvement over time—which to some extent, reflects that the efficacy of conventional treatment cannot be ignored.

A third reason may be related to the specific conditions of certain participants. Some participants in the study group (*n* = 8) failed to attend the entire 10 group music therapy due to reasons such as early discharge, which somewhat attenuated the average efficacy of participants in the study group. Meanwhile, due to the possibility of mutual communication between the two groups of participants and Zelen’s trial design (which is closer to the real-world clinical situation), the participants in the control group were not strictly blinded. Under these conditions, participants in the control group may have gleaned relevant content about the group music therapy from the study group’s participants. This narrows the difference in efficacy between the two groups. In addition, as mentioned above, numerous participants may conceal their real situation in order to be discharge. The reasons for such eagerness may include objective factors such as limited economic resources and maladjustment to the closed inpatient environment. It may also include subjective factors such as blind self-confidence in recovery status and eagerness caused by active suppression of their desire for alcohol. In addition, implementing treatment close to traditional Chinese festivals may be less effective, since some participants may be less at ease about being hospitalized for treatment.

## Strength and Limitations

This study is a useful attempt to apply intensive group music therapy based on emotion-regulation skills to male AD inpatients, providing a theoretical and practical basis for the development of related research and treatment in the future. This study is one of the few real-world clinical studies designed using Zelen’s approach in the field of psychotherapy. Of course, this study also has some limitations: small sample, lack of biological and other objective indicators for the assessment of efficacy, and no long-term follow-up at 6 months and longer.

## Conclusion

The study shows that intensive group music therapy based on emotion-regulation skills is acceptable, feasible with the potential for efficacy, for male AD patients in closed inpatient settings. Participants gave very positive feedback to group music therapy and expressed their willingness to continue to participate in such group therapy. This group music therapy has some benefits for relieving anxiety, improving sleep quality, improving treatment participation and preserving AD patients, although the efficacy advantage was not significant in our study due to objective factors such as sample size and treatment length. Nevertheless, our study appears to be the first report of an intensive group music therapy intervention trial based on emotion-regulation skills, and it justifies further research into this promising treatment, preferably with larger samples and longer follow-up.

## Data Availability Statement

The raw data supporting the conclusions of this article will be made available by the authors, without undue reservation.

## Ethics Statement

The studies involving human participants were reviewed and approved by the Ethics Committee of the Shandong Mental Health Center (2020R22). The patients/participants provided their written informed consent to participate in this study.

## Author Contributions

Both the authors are responsible for the content and preparation of this manuscript. YH and XC designed the study and performed the statistical analysis. YH conducted a literature search and provided a summary of previous studies and wrote the first draft of the manuscript. Both the authors participated in and approved the final manuscript.

## Conflict of Interest

The authors declare that the research was conducted in the absence of any commercial or financial relationships that could be construed as a potential conflict of interest.

## Publisher’s Note

All claims expressed in this article are solely those of the authors and do not necessarily represent those of their affiliated organizations, or those of the publisher, the editors and the reviewers. Any product that may be evaluated in this article, or claim that may be made by its manufacturer, is not guaranteed or endorsed by the publisher.
